# High-throughput strategy for targeting MDM2 in uveal melanoma to reverse radiation therapy resistance

**DOI:** 10.1038/s41420-026-02970-x

**Published:** 2026-04-11

**Authors:** Qi Zhu, Xi Gong, Sirong Zhang, Jiaqi Zhang, Hongtao Yan

**Affiliations:** Department of Ophthalmology, The Second Hospital of Jilin University, Changchun, China

**Keywords:** Biotechnology, Diseases

## Abstract

Uveal melanoma (UM) presents a formidable clinical challenge due to its marked resistance to radiotherapy. In this study, an integrative strategy combining machine learning models with high-throughput screening platforms was employed to identify novel small-molecule inhibitors targeting MDM2, with the aim of overcoming this intrinsic resistance. Transcriptome sequencing and machine learning analysis identified MDM2 as a critical gene associated with UM radiotherapy resistance. Integration of single-cell RNA sequencing data revealed key cells contributing to this resistance. In vitro experiments demonstrated that the MDM2 inhibitor SAR405838 effectively increased radiosensitivity in resistant UM cells by modulating p53 activation, suppressing cell migration and invasion, and inducing DNA damage and apoptosis. This novel approach offers a promising therapeutic strategy for combating UM resistance to radiation therapy.

## Introduction

Uveal melanoma (UM) is a prevalent malignant ocular tumor, marked by a painless onset, rapid progression, and treatment difficulties due to its invasive nature [1, 2]. As UM primarily affects individuals of working age, effective management is essential for preserving quality of life and improving survival outcomes [3–5]. However, resistance to conventional treatments like radiotherapy poses a major challenge, limiting therapeutic efficacy [6–8]. The development of novel therapeutic strategies is required to improve outcomes in UM.

Radiotherapy resistance continues to challenge clinical management of UM, with conventional approaches often yielding suboptimal results [9, 10]. Advances in machine learning algorithms and high-throughput technologies have opened new avenues for exploring the molecular mechanisms underlying resistance and identifying precise therapeutic targets [11–13]. Previous studies have shown that the MDM2 inhibitor Navtemadlin can suppress melanoma growth in mice and enhance the efficacy of radiotherapy [14]. Inhibition of MDM2 presents a promising therapeutic opportunity for glioblastoma, either as a monotherapy or in combination with chemotherapy and/or other targeted agents, and numerous small-molecule MDM2 inhibitors are currently undergoing clinical evaluation [15]. Moreover, antagonists of MDM2 have been reported to enhance the radiosensitivity of wild-type p53 esophageal squamous cell carcinoma [16]. The present study utilizes these technologies to identify small-molecule inhibitors targeting MDM2, aiming to activate the p53 signaling pathway, enhance the DNA damage response, and overcome resistance to radiotherapy in UM, thereby improving therapeutic efficacy [17–19].

In this study, a series of experiments was designed to elucidate the mechanisms of radiotherapy resistance in UM [20–22]. Using transcriptome sequencing technology, we conducted an in-depth analysis of UM tissues and employed machine learning algorithms to identify key genes involved in radiotherapy resistance, thereby revealing potential therapeutic targets [23]. In vitro assays were performed to evaluate the impact of a small molecule inhibitor targeting MDM2 on radiotherapy-resistant UM cells. The mechanism of action was evaluated through multiple indicators, including the Cell Counting Kit-8 (CCK-8) assay to measure cell viability and immunofluorescence staining for γ-H2AX expression [24, 25].

The primary objective of the study was to integrate machine learning algorithms with high-throughput screening to identify MDM2-targeted small-molecule inhibitors that activate p53 signaling, enhance the DNA damage response, and reverse resistance to radiotherapy in UM. Experimental findings confirmed that MDM2 inhibition significantly increased radiosensitivity in UM cells, suppressed migration and invasion, and induced DNA damage and apoptosis. These results provide a promising therapeutic strategy for improving treatment efficacy in UM. The proposed approach offers a foundation for the development of novel targeted therapies with strong clinical potential to improve patient survival and quality of life.

## Results

### Screening of key gene MDM2 associated with radiotherapy resistance in UM

**Fig. 1:**
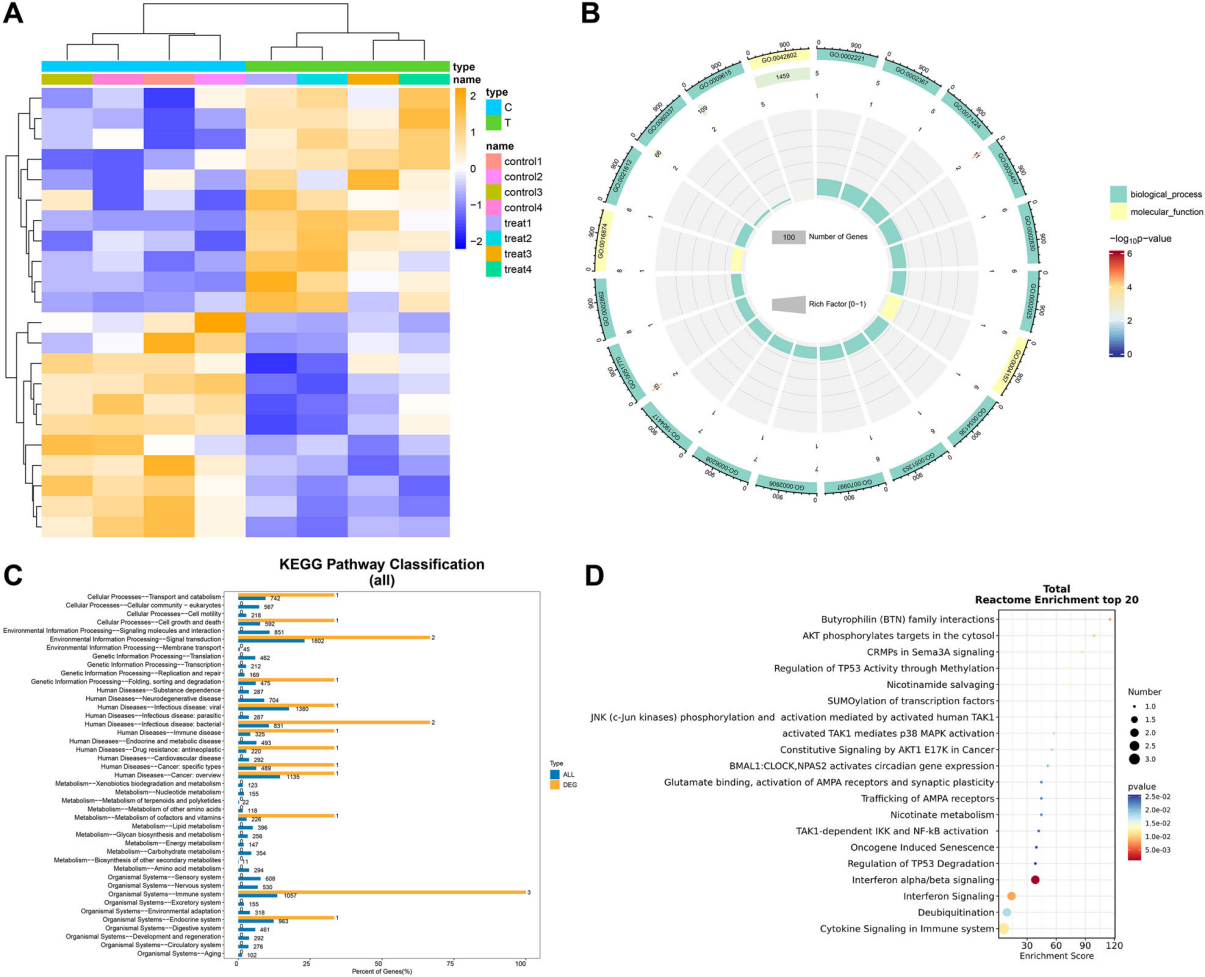
Transcriptomic analysis to identify radiotherapy resistance in UM. **A** Differential expression heatmap analysis of transcriptome sequencing of UM radiotherapy-resistant cells and sensitive cells; **B** GO enrichment analysis of DEGs; **C** KEGG enrichment analysis of DEGs; **D** Reactome enrichment analysis of DEGs.

To further investigate the core genes influencing radiotherapy resistance in UM, we performed transcriptome sequencing of UM radiotherapy-resistant and -sensitive cells. Differential expression analysis identified 22 differentially expressed genes (DEGs) (Fig. 1A). GO enrichment indicated involvement of DEGs in cellular response to interferon-alpha and negative regulation of the p53-mediated DNA damage response. Kyoto Encyclopedia of Genes and Genomes (KEGG) analysis linked the DEGs to pathways related to cell growth and death, transport and catabolism, and signal transduction. Reactome pathway analysis further associated the DEGs with interferon alpha/beta signaling and regulation of TP53 degradation (Fig. 1B–D).

**Fig. 2:**
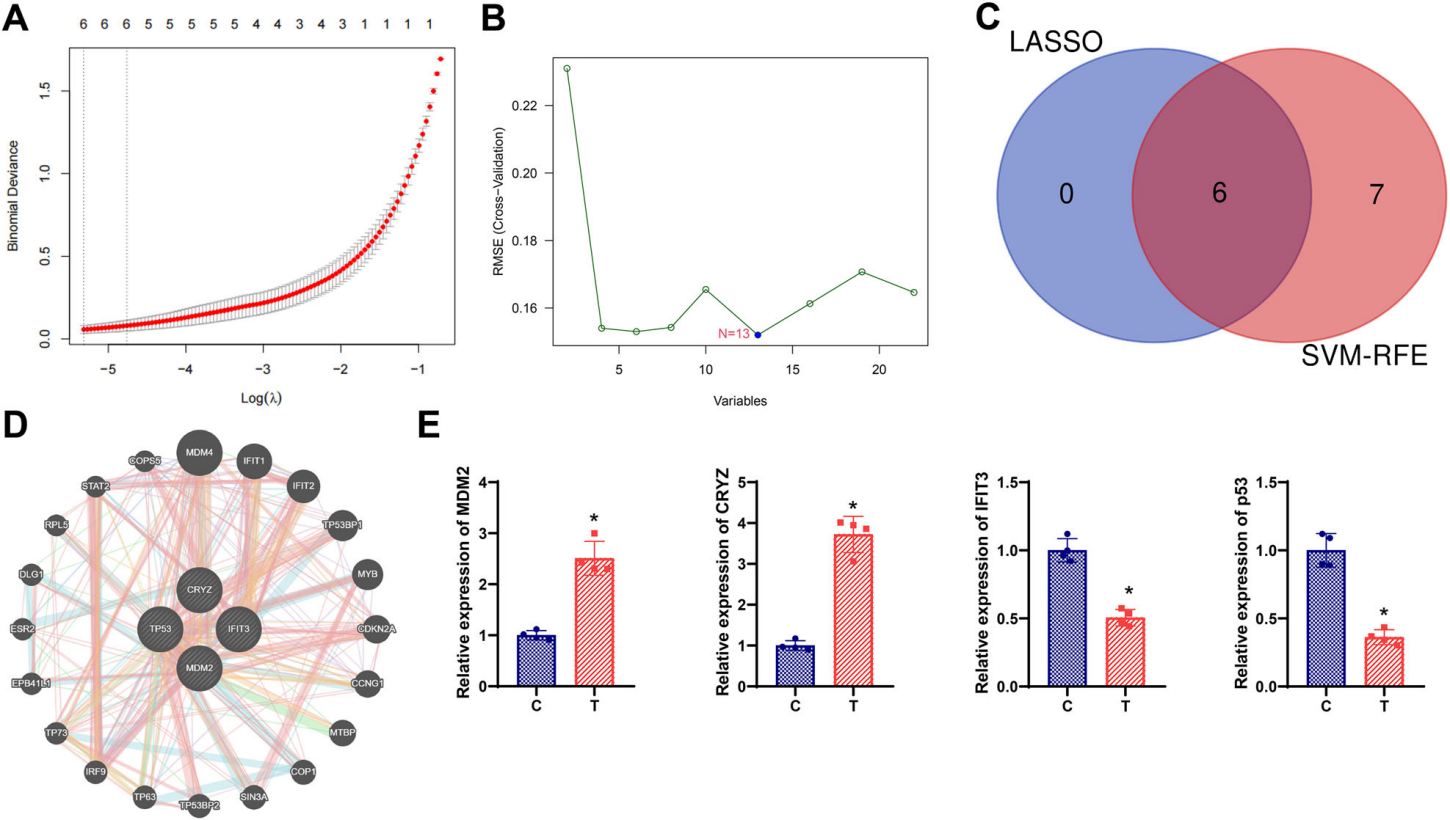
Identification of core genes associated with UM radiotherapy resistance. **A** Graph illustrating the selection process for the cross-validation parameter λ; **B** Line graph showing feature selection through cross-validation using VM-RFE algorithm; **C** Venn analysis to identify common feature genes obtained by screening with LASSO and SVM-RFE algorithms; **D** PPI network of MDM2, CRYZ, IFIT3, and p53; **E** Comparison of the expression levels of MDM2, CRYZ, IFIT3, and p53 in radiotherapy-resistant cells compared to radiotherapy-sensitive cells. * indicates that compared to the control group, *p* < 0.05.

To refine the list of resistance-associated genes, least absolute shrinkage and selection operator (LASSO) regression was applied to the 22 DEGs. Based on the L1 regularization method, changes in regression coefficients were evaluated (Fig. 2A). The penalty parameter α was set to 1, and tenfold cross-validation was used to optimize model performance. The model achieved the lowest root mean squared error with six variables, narrowing the selection to four candidate DEGs.

To mitigate potential bias caused by sample imbalance, SVM–RFE was also employed (Fig. 2B). This method identified optimal feature combinations among the candidate DEGs. A Venn diagram analysis comparing the outputs of LASSO and SVM–RFE revealed six overlapping genes: MDM2, USP32P2, CRYZ, MIR221, IFIT3, and TP53 (Fig. 2C). After excluding non-coding RNAs, MDM2, CRYZ, IFIT3, and TP53 were retained as final candidate markers. A protein–protein interaction (PPI) network was subsequently constructed using the GeneMANIA database to explore their functional relationships (Fig. 2D).

Expression analysis showed that MDM2 and CRYZ were upregulated, while IFIT3 and TP53 were downregulated in radiotherapy-resistant cells compared to sensitive cells, with MDM2 exhibiting the most pronounced differential expression (Fig. 2E). Prior studies have demonstrated that MDM2 suppresses p53 transcriptional activity, thereby inhibiting downstream targets such as cell cycle arrest genes [26]. These results imply that MDM2 may function as a central regulator in UM radiotherapy resistance.

### Quality control, filtering, and PCA of scRNA-seq data on radiotherapy-resistant UM tissue

**Fig. 3:**
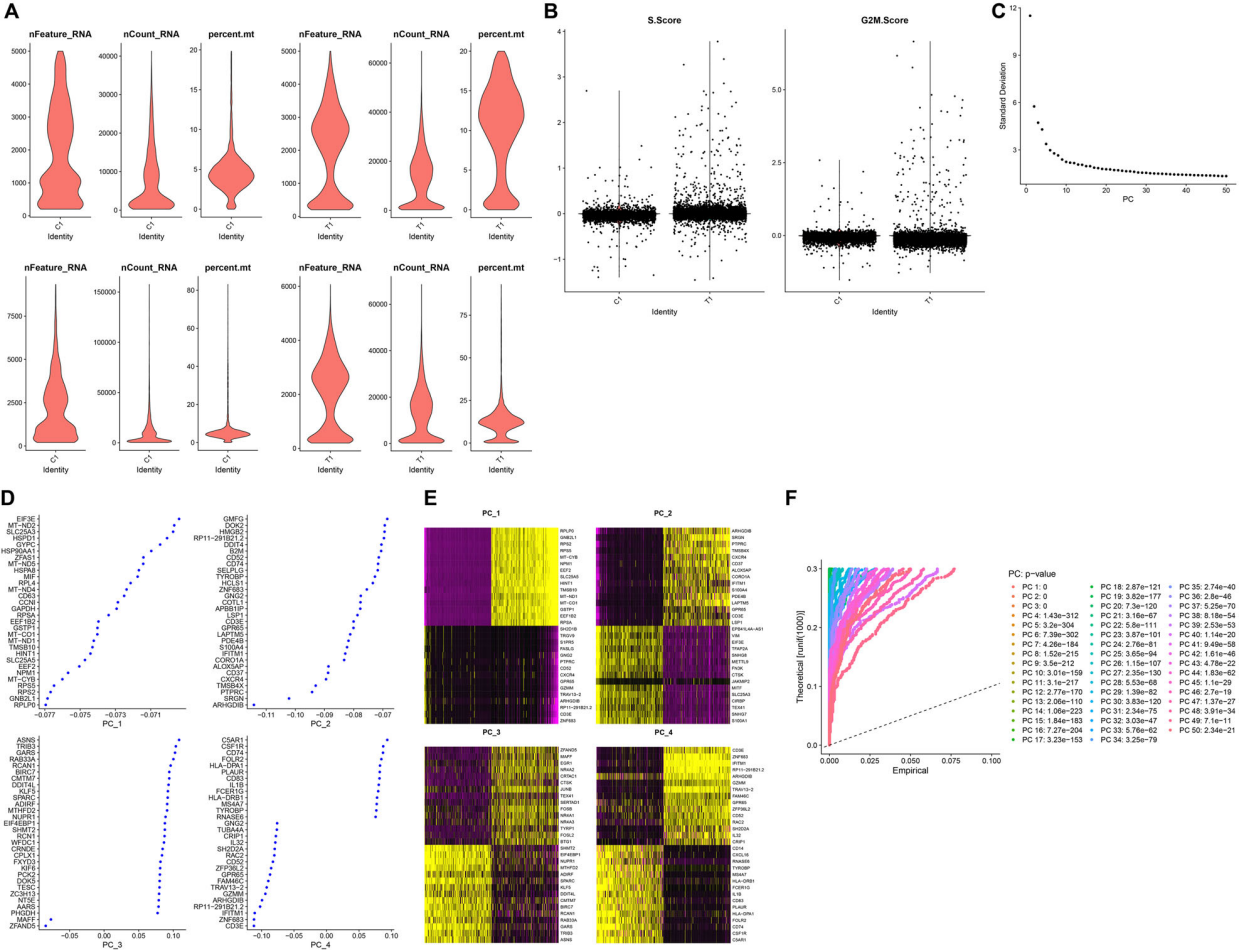
Quality control, filtering, and PCA of scRNA-seq data. **A** Quality control assessment of each cell in the scRNA-seq data, with three scatter plots displaying the quantities of nFeature_RNA, nCount_RNA, and percent.mt per cell; **B** Calculation of S.Score and G2M.Score for scRNA-seq data; **C** Results of dimensionality reduction analysis where each point represents a single cell; **D** Scatter plot showing expression levels of feature genes in the top 4 PCs from PCA analysis; **E** Heatmap illustrating expression levels of feature genes in the top 4 PCs from PCA analysis, with yellow indicating upregulation and purple indicating downregulation; **F** The *p*-values of the 50 PCs obtained from PCA of cells from different sample sources; *n*(*T*) = 2.

We utilized the “Seurat” package in the R software to analyze the single-cell dataset GSE139829. Following standard quality control procedures, including filtering and normalization, the distribution of RNA features per cell was obtained (Fig. 3A). Cell cycle phase scores (S.Score and G2M.Score) were calculated to evaluate cell cycle states (Fig. 3B). The top 2000 highly variable genes were identified based on expression variance and selected for downstream analysis (Fig. 3C), indicating significant transcriptional heterogeneity in UM tissues.

Principal component analysis (PCA) was then performed on the selected variable genes to reduce dimensionality and facilitate subsequent clustering via t-stochastic neighbor embedding (t-SNE) (Fig. 3C). Representative expression patterns of the top four principal components (PCs) were visualized using heatmaps and bubble plots (Fig. 3D, E). Key PCs were further identified using the JackStrawPlot function, which compares the distribution of PC-associated *p*-values to a null distribution. PCs with significantly lower *p*-values (solid lines above the dashed threshold) were considered informative for capturing transcriptomic variation. A total of 50 PCs were extracted and retained for downstream t-SNE clustering and cell-type annotation (Fig. 3F), supporting the effectiveness of PCA in resolving cell subpopulations within UM tissues.

### Cell clustering identifies cells related to UM radiotherapy resistance and marker genes

**Fig. 4:**
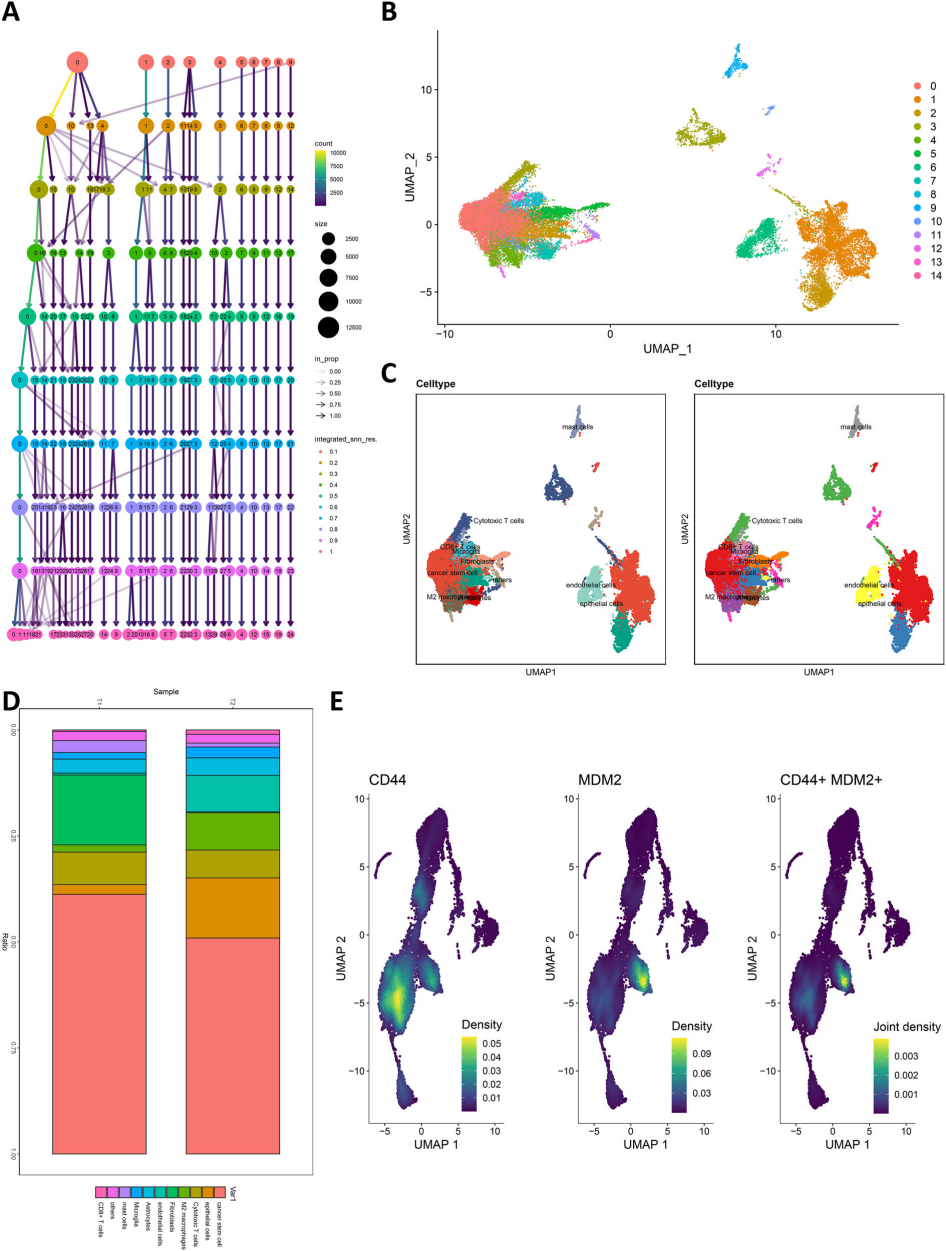
Cell clustering analysis of scRNA-seq data. **A** Find Clusters function selects the optimal number of clusters; **B** t-SNE clustering analysis clusters cells into 15 cell clusters; **C** SingleR annotates the 15 cell clusters into 10 cell types; **D** Proportion of the 10 annotated cell types across different UM sample groups; **E** Co-expression region plot of CD44 and MDM2 in the sc-RNA sequencing data. *n*(*T*) = 2.

To classify cell populations, we first used the Find Clusters function to select the optimal number of clusters (Fig. 4A), followed by clustering all cells into 14 cell clusters using t-SNE analysis (Fig. 4B). Cell types within each cluster were annotated using the Bioconductor package “SingleR,” which identified major populations including cancer stem cells (CSCs), epithelial cells, cytotoxic T cells, M2 macrophages, fibroblasts, endothelial cells, astrocytes, microglia, mast cells, and CD8⁺ T cells (Fig. 4C). Proportional analysis of cell types showed that CSCs were the dominant population in both UM samples (T1 and T2), while other cell types such as epithelial cells, cytotoxic T cells, M2 macrophages, and fibroblasts were also widely distributed throughout the tumor tissue, reflecting the heterogeneity of the tumor microenvironment (Fig. 4D).

Among the identified cell populations, CSCs exhibit strong DNA damage repair capabilities, particularly in response to radiation-induced double-strand breaks [27]. Co-expression analysis revealed a notable spatial overlap between the stemness marker CD44 and MDM2 (Fig. 4E), suggesting that CSCs may participate in suppressing the DNA damage response through MDM2-mediated inhibition during radiotherapy resistance in UM. Transcriptomic profiling from the The Cancer Genome Atlas Uveal Melanoma (TCGA-UVM) cohort further demonstrated significant positive correlations between CD44 and key components of the DNA damage response pathway, including double-strand break sensors (ATM, ATR), checkpoint regulator (CHEK1), homologous recombination repair genes (RAD51, BRCA1, BRCA2), single-strand break repair enzyme (PARP1), stress response mediator (GADD45A), and the p53 pathway regulator MDM2 (Fig. S1). These results further support the potential role of CSCs in mediating radiotherapy resistance.

### Reversal of radiotherapy resistance in UM cells by MDM2 inhibitors

Bioinformatics analyzes suggested that MDM2 contributes to radiotherapy resistance in UM by inhibiting the DNA damage response pathway. Previous research has demonstrated that MDM2 promotes p53 degradation via ubiquitination, thereby reducing p53 protein stability and enhancing cell proliferation and drug resistance in colorectal cancer [28]. MDM2 negatively regulates p53, contributing to the progression of diffuse large B-cell lymphoma (DLBCL) [29]. Alterations in the p53 signaling pathway are associated with the occurrence of conjunctival and corneal tumors, retinoblastoma, UM, and intraocular melanoma, where the biological activity of p53 extends beyond cell cycle arrest to regulating homeostasis, DNA repair, cell apoptosis, and senescence. Therefore, loss-of-function p53 mutations in p53 are widely recognized as key drivers of tumorigenesis [30].

**Fig. 5:**
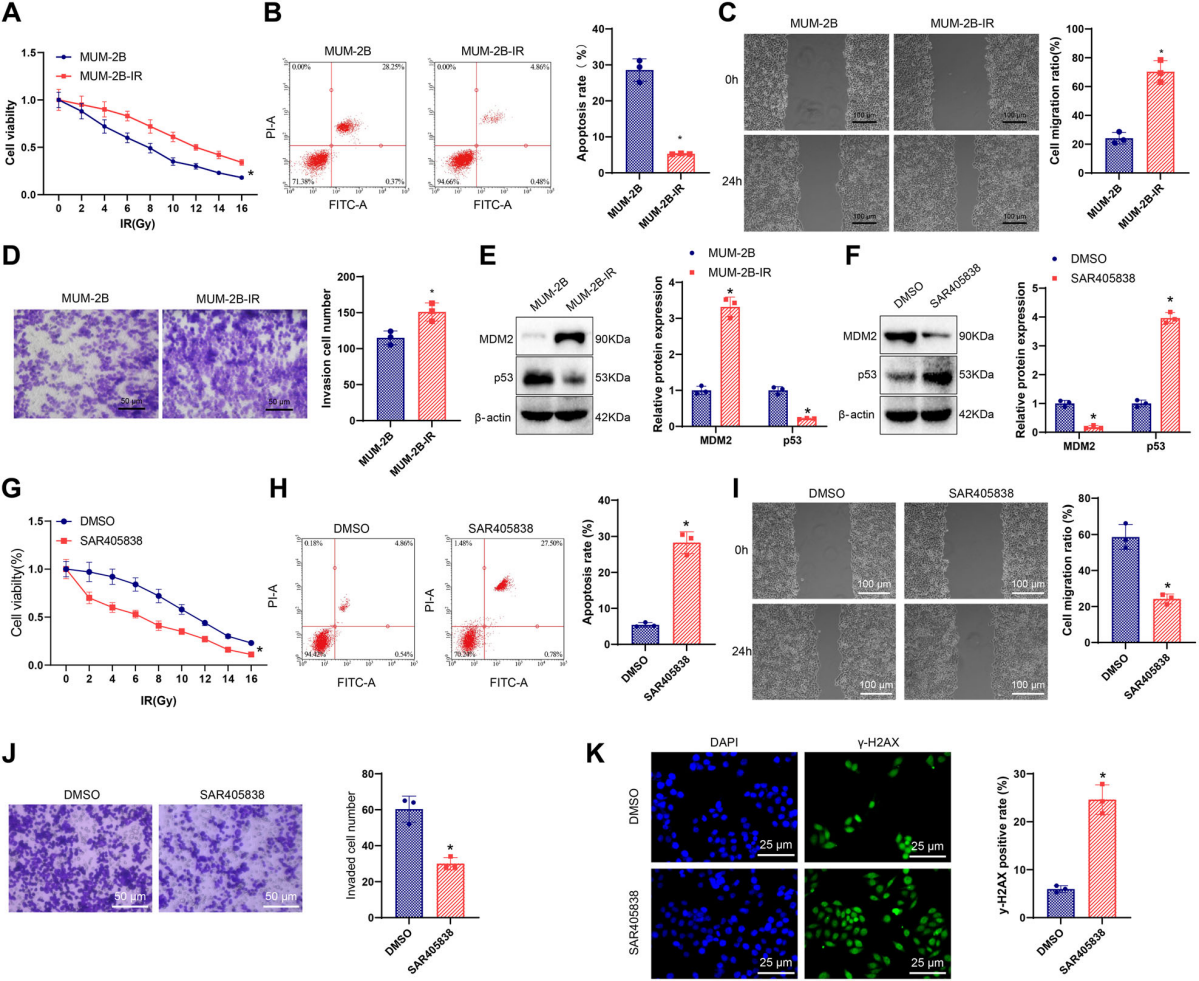
Reversal of radiotherapy resistance in UM cells by MDM2 inhibitors. **A** Cellular viability of MUM-2B and MUM-2B-IR cells at different radiation doses assessed by CCK-8 assay; **B** Flow cytometry analysis of apoptosis in MUM-2B and MUM-2B-IR cells; **C** Wound healing assay assessing the migration capacity of MUM-2B and MUM-2B-IR cells; **D** Transwell invasion assay evaluating the invasive ability of MUM-2B and MUM-2B-IR cells; **E** Expression levels of MDM2 and p53 in MUM-2B and MUM-2B-IR cells detected by Western blot; **F** Expression levels of MDM2 and p53 in MUM-2B-IR cells in each group examined by Western blot; **G** Cell viability of MUM-2B-IR cells at different radiation doses measured by CCK-8 assay; **H** Apoptosis rate of MUM-2B-IR cells in each group determined by flow cytometry; **I** Migration capability of MUM-2B-IR cells in each group evaluated by wound healing assay; **J** Invasion ability of MUM-2B-IR cells in each group assessed by Transwell assay; **K** Expression of γ-H2AX in MUM-2B-IR cells in each group detected by immunofluorescence staining. *Indicates *p* < 0.05 compared to the MUM-2B group or DMSO group, with cell experiments repeated three times.

To validate the role of the MDM2/p53 axis in UM radiotherapy resistance, a resistant MUM-2B-IR cell line was established through repeated irradiation. These cells displayed hallmark features of resistance, including increased viability, enhanced migration and invasion, and decreased apoptosis relative to parental controls (Fig. 5A–D). Consistent results were also observed in the OMM2.3 cell line (Fig. S2A, D).

Western blot analysis revealed elevated MDM2 protein levels and reduced p53 expression in both MUM-2B-IR and OMM2.3-IR cells (Figs. 5E and S2E). These findings provide mechanistic evidence that MDM2 upregulation and concurrent p53 suppression are central to the development of radiotherapy resistance in UM.

A series of treatments with an MDM2 inhibitor (SAR405838) was applied to the radiotherapy-resistant cells. Western blot analysis demonstrated a marked decrease in MDM2 protein levels and a concomitant increase in p53 expression following SAR405838 treatment, relative to the dimethyl sulfoxide (DMSO) control group (Figs. 5F and S2F).

Cell viability assays using CCK-8 revealed a significant reduction in proliferation in SAR405838-treated cells compared to DMSO-treated controls, indicating restored sensitivity to radiotherapy (Figs. 5G and S2G). Flow cytometry results indicated that the MDM2 inhibitor SAR405838 induced apoptosis in the radiotherapy-resistant MUM-2B-IR cells (Figs. 5H and S2H). Additionally, wound healing and Transwell assays confirmed that SAR405838 markedly suppressed the migratory and invasive capacities of MUM-2B-IR cells (Figs. 5I, J and S2I, J).

Immunofluorescence staining for γ-H2AX, a marker of DNA double-strand breaks, revealed increased expression in SAR405838-treated cells, suggesting activation of the DNA damage response pathway (Figs. 5K and S2K).

These results collectively suggest that the MDM2 inhibitor can suppress the radiotherapy resistance, cellular migration, and invasion capabilities, and promote apoptosis and DNA damage in radiotherapy-resistant UM cells.

## Discussion

UM is one of the most prevalent intraocular malignancies and remains difficult to treat due to its intrinsic resistance to radiotherapy [31]. The aim of this study is to utilize machine learning algorithms and high-throughput techniques to screen for small molecule inhibitors targeting MDM2 to enhance the sensitivity of UM cells to radiotherapy. In the methodology, the study employed a mouse UM radiotherapy-resistant model, used transcriptome sequencing and machine learning to identify core genes, and further employed single-cell sequencing to isolate core cells. The study ultimately validated the reversal effect of MDM2 inhibitors on UM [32].

Previous studies have demonstrated that MDM2, the core gene identified in this study, contributes to oncogenesis by promoting genomic instability, tumor metastasis, and epithelial–mesenchymal transition [33]. In addition, MDM2 acts as an E3 ubiquitin ligase that degrades the tumor suppressor protein p53, thereby regulating cell proliferation and apoptosis—critical processes in cancer progression [34–36]. Moreover, the relationship between CSCs, p53, and MDM2 has been increasingly recognized. For instance, MDM2 promotes sustained ubiquitination and degradation of the androgen receptor (AR) in prostate CSCs, thereby enhancing self-renewal, proliferation, and stemness gene expression [37]. MDM2 also physically interacts with chromatin-bound EZH2, increasing H3K27me3 and H2AK119ub, thereby maintaining stemness and supporting CSC survival [38]. These findings support our observation of co-expression between MDM2 and CD44 (a CSC marker) in UM. Clinically, MDM2 is frequently overexpressed or amplified in various cancer types and is associated with poor prognosis [39, 40]. As a result, targeting MDM2 is considered a promising strategy for cancer prevention and treatment. Several MDM2-targeting therapeutic approaches—including small-molecule inhibitors, protein destabilizers, and proteolysis-targeting chimeras—have been actively investigated [18, 41]. Although phase I clinical trials of most MDM2 inhibitors have shown limited efficacy [42], numerous candidates are currently undergoing phase II/III trials for p53 wild-type cancers [43, 44], offering precedent and optimism for the continued development of MDM2-targeted therapies in UM.

Unlike conventional strategies, the present study offers a forward-looking and efficient approach to drug screening [45–47]. While prior studies often relied on single-pathway or low-throughput screening methods, this work integrates machine learning algorithms with high-throughput technologies to improve both the accuracy and speed of compound discovery [48]. This innovative screening approach holds great promise in future cancer treatment research and introduces novel perspectives to the field of drug screening [49].

Through transcriptomic and single-cell sequencing methods, this study elucidated the significance of MDM2 in UM’s resistance to radiation therapy. Elevated MDM2 expression was inversely correlated with p53 protein levels, suggesting that MDM2 is a key driver of resistance. These observations are consistent with earlier findings and reinforce the importance of MDM2 as a therapeutic target in UM [50, 51].

In vitro experiments revealed that the MDM2 inhibitor significantly increased the sensitivity of radiation-resistant cells, inhibited their migration and invasion capabilities, and promoted DNA damage and apoptosis. These effects support a novel therapeutic mechanism for MDM2 inhibitors in UM, distinct from prior reports that focused primarily on tumor growth suppression [15, 52]. Although the overall findings align with existing literature, the differences in screening methodology and biological focus provide new insight into resistance mechanisms and therapeutic potential in UM.

**Fig. 6:**
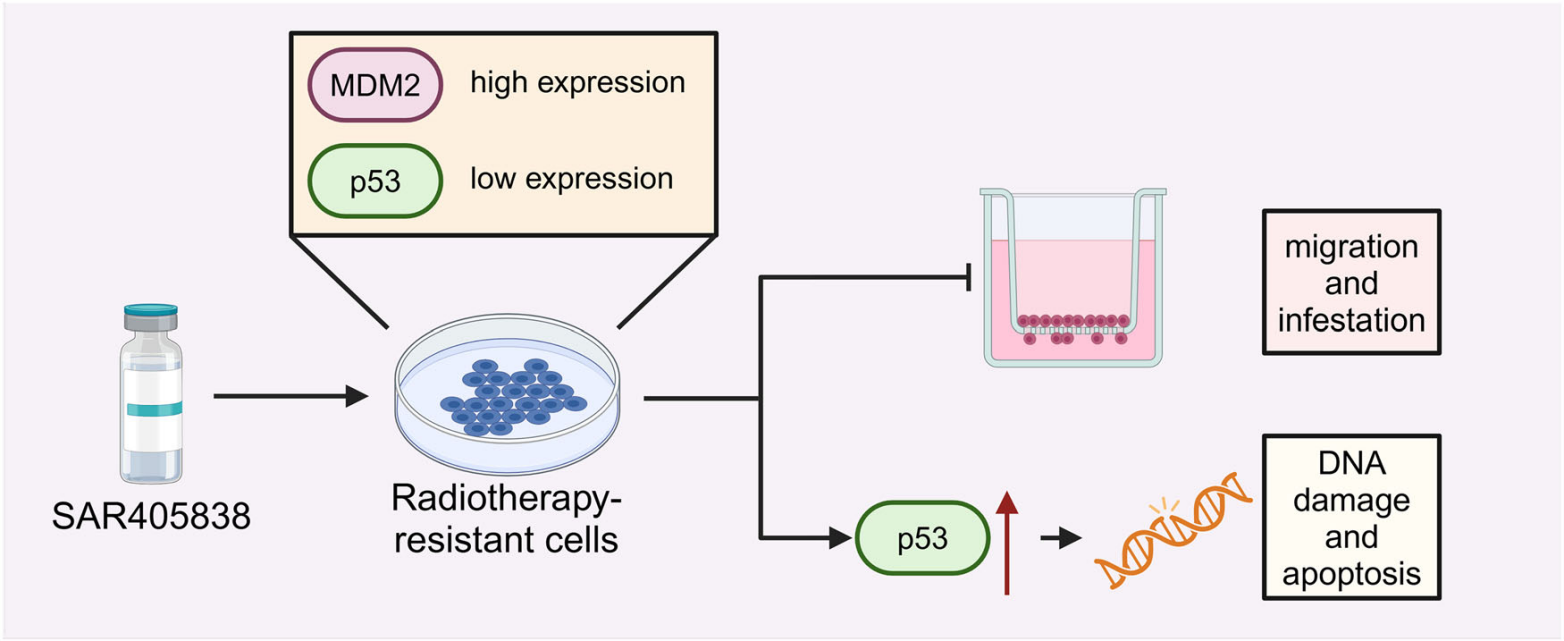
Mechanisms of MDM2 inhibitor reversing UM radiation therapy resistance (Created with BioRender.com).

In summary, this study successfully screened small molecule inhibitors targeting MDM2 through the integration of machine learning algorithms and high-throughput techniques. This activation of p53 enhances DNA damage response and reverses radiotherapy resistance in UM (Fig. 6). Through transcriptomic and proteomic data analysis, we identified genes and proteins with differential expression before and after radiotherapy, elucidating the biological processes and molecular mechanisms related to radiotherapy resistance. The application of deep generative learning models has improved the efficiency and accuracy of screening small molecule inhibitors. In vitro experiments demonstrated that MDM2 inhibitors effectively reduced the viability of resistant UM cells and restored p53 function through induction of apoptosis.

The scientific and clinical value of this work lies in establishing an innovative strategy for overcoming radiotherapy resistance in UM by targeting MDM2. The approach not only enhances radiosensitivity by reactivating p53 signaling and DNA damage pathways but also offers a promising therapeutic avenue for clinical translation. These findings provide both experimental validation and a solid theoretical framework for the future development of MDM2-targeted therapies in UM treatment.

However, this study also has certain limitations. First, the diversity of current bioinformatics tools and sequencing platforms may influence the interpretation of results across datasets. Our study lacked sequencing data from clinical UM samples to validate the bioinformatic findings. Moreover, the limited availability of well-characterized CSC markers in UM also hindered the precision of single-cell annotation. Future work should incorporate additional or UM-specific markers to confirm the involvement of CSCs in DNA damage responses. Second, conducting in vivo experiments with MDM2 inhibitors is essential to validate their preclinical therapeutic efficacy. The current lack of in vivo data represents a clear limitation of this study, as in vitro systems cannot fully replicate the complexity of the tumor microenvironment or systemic physiological responses. In future studies, the radioresistant cell lines established in this work will be used to generate subcutaneous and orthotopic xenograft models in immunodeficient mice. Four experimental groups will be established: a control group, a radiotherapy group, an inhibitor group, and a combination treatment group. These models will be used to systematically evaluate the effects of SAR405838 on tumor growth inhibition and mouse survival. Tumor tissue analyzes will be performed to verify in vivo activation of the MDM2–p53 pathway, as well as DNA damage and apoptosis induction, while also providing preliminary safety assessments of the compound. In subsequent investigations, the parental radiosensitive cell lines (MUM-2B and OMM2.3) will be included as essential controls. Markers such as γ-H2AX will be examined to comprehensively characterize the dynamic process from “sensitivity” to “resistance” and ultimately to “reversal.” Moreover, while the experimental methods used in the study are reliable, the complexity of clinical scenarios may impact actual treatment outcomes, necessitating more preclinical and clinical trials to confirm the feasibility and safety of its application.

Looking ahead, the therapeutic potential of MDM2 inhibitors warrants exploration beyond UM. Structural optimization and pharmacokinetic enhancement may further improve their clinical efficacy and safety profiles. Cross-disciplinary collaborations will be essential to accelerate clinical translation and deliver tangible therapeutic benefits to patients.

## Materials and methods

### Establishment of UM cell culture and radiotherapy resistance model

The UM cell line (MUM-2B, purchased from Shanghai Yagene Biotechnology Co., Ltd, YS206C) and OMM2.3 (purchased from Guangzhou Cellcook Biotech Co., Ltd, CC1809) were cultured in RPMI-1640 medium (Gibco, 11875093, USA) containing 10% fetal bovine serum (FBS; Gibco, 10100147, USA). Cells were maintained at 37 °C in a 5% CO_2_ atmosphere. To establish a radiotherapy-tolerant cell line, the above cells were exposed to 2 Gy of ionizing radiation twice per week (dose rate: 1 Gy/min; irradiation duration: 2 min) for 3 consecutive months. The resulting radiation-tolerant cell lines were designated as MUM-2B-IR and OMM2.3-IR. Radiotherapy tolerance was evaluated using the CCK-8 assay [53]. The radiotherapy-resistant cells were then subjected to the following treatments and grouped as follows: DMSO group (vehicle control) and SAR405838 group (treatment with MDM2 inhibitor SAR405838 (Sigma, SML2772) at 2 µM for 1 h) [54].

### Transcriptome sequencing analysis

Transcriptome sequencing was performed on both drug-resistant and drug-sensitive tumor cell lines. Differential analysis was performed using the R package “limma,” with a cutoff criteria of |log2FC| > 1 and a significant *P*-value < 0.05 to define DEGs. Visualization of DEGs was achieved by generating volcano plots using the “ggplot2” package, while hierarchical clustering heatmaps were created using the “heatmap” package in R [55, 56].

### Functional enrichment analysis of DEGs

Gene Ontology (GO), KEGG, and Reactome pathway enrichment analyzes were carried out using the “ClusterProfiler” package in R, with a significance threshold set at *P* < 0.05. GO analysis included three major categories: biological processes (BP), molecular functions (MF), and cellular components (CC). Enrichment results provided functional insights into cellular pathways and molecular mechanisms regulated by candidate target genes [57].

### Least absolute shrinkage and selection operator (LASSO) regression analysis

LASSO regression was applied to evaluate the relationship between dependent and independent variables using the “glmnet” function in R Studio (version 4.0.2). Candidate gene expression matrices were input into the model, with the penalty parameter α fixed at 1. Tenfold cross-validation was used to determine the optimal value of the regularization parameter *λ* and identify the most predictive gene subset. In the LASSO model, m denotes the number of samples, *k* represents the number of variables, and *λ* indicates the regularization coefficient used to minimize overfitting and enhance model performance [58].

$$\frac{1}{2m}\left[\mathop{\sum }\limits_{i=1}^{m}{\left({{\mathrm{h}}}_{{\rm{\theta }}}\left({x}^{\left(i\right)}\right)-{y}^{\left(i\right)}\right)}^{2}+\lambda \mathop{\sum }\limits_{j=1}^{k}|{w}_{j}|\right]$$

### Support vector machine - recursive feature elimination (SVM-RFE) analysis

The recursive feature elimination (RFE) algorithm was applied using the “e1071” and “caret” packages in R (version 4.0.2) to identify an optimal subset of genes. Gene expression levels were used as features, while clinical variables served as categorical outcomes. A support vector machine (SVM) with a linear kernel was employed to classify patient groups, and the RFE procedure was used to iteratively eliminate non-informative features and retain the most predictive gene signatures [59].

### Venn diagram analysis

Candidate genes were identified using Venn diagram analysis, which was performed with the Draw Venn Diagram tool [60].

### Single-cell sequencing data analysis

Single-cell RNA-seq (scRNA-seq) data of uveal melanoma were obtained from the GEO (https://www.ncbi.nlm.nih.gov/geo/) under the accession number GSE139829. Samples GSM4147093 and GSM4147094 were analyzed using the “Seurat” package (version 4.0.2) in R (version 4.0.2) [61]. These two samples were selected based on their higher sequencing depth, sufficient cell numbers, favorable quality-control metrics, and consistent technical conditions; other samples were excluded due to low cell counts. Raw data were first subjected to quality control, with filtering thresholds set as follows: nFeature_RNA > 500, nCount_RNA > 1000, nCount_RNA < 20000, and percent.mt < 10. Batch effects between samples were corrected using Canonical Correlation Analysis, followed by data normalization using the LogNormalize function. PCA was then performed, and the optimal number of clusters was determined using the FindClusters function. Dimensionality reduction and visualization were conducted using t-SNE. Cell-type annotation was performed with the Bioconductor/R package “SingleR,” using the MouseRNAseqData reference dataset to identify marker genes and assign cell identities [62, 63].

### Western blot analysis

Total cellular proteins were extracted using radioimmunoprecipitation assay lysis buffer (P0013B, Beyotime, Shanghai, China) supplemented with phenylmethylsulfonyl fluoride and quantified using the bicinchoninic acid protein assay kit (23225, Thermo Fisher Scientific, Rockford, IL, USA). Equal amounts of protein (50 µg) were mixed with 2× sodium dodecyl sulfate (SDS) loading buffer, denatured at 100 °C for 5 min, and subjected to SDS-polyacrylamide gel electrophoresis (SDS-PAGE). Separated proteins were transferred onto polyvinylidene fluoride membranes using a wet transfer system. Membranes were blocked with 5% skimmed milk in Tris-buffered saline with 0.1% Tween-20 (TBST) for 1 h at room temperature and incubated overnight at 4 °C with primary antibodies against MDM2 (ab259265, Abcam, UK; 1:1000), p53 (ab154036, Abcam, 1:1000), and β-actin (ab5694, Abcam, 1:1000) overnight at 4 °C. After washing with Tris-buffered saline with 0.1% Tween 20 (TBST) three times for 10 min each, the membrane was incubated with an HRP-conjugated secondary antibody goat anti-rabbit IgG H&L (HRP) (ab97051, Abcam, Cambridge, UK) at a 1:2000 dilution for 1 h. Following TBST washes, the membrane was placed on a clean glass plate. An equal amount of solution A and B from the Pierce™ ECL detection kit (32209, Thermo) was mixed in the darkroom and then added to the membrane. Imaging was performed using the Bio-Rad imaging system (ChemiDoc™ XRS+, BIO-RAD, USA) [64]. Original WB images can be found at the supplementary information.

### CCK-8

Cell proliferation experiments were conducted using the CCK-8 assay kit (CA1210, Solarbio, Beijing, China). Following cellular treatment, 10 µl of CCK-8 reagent was added to each well and incubated at 37 °C for 3 h. The absorbance values at 450 nm were measured with a Synergy H1 microplate reader (BioTek, USA) to quantify cell viability [65].

### Flow cytometry for apoptosis detection

Apoptotic cells were detected using the Annexin V–fluorescein isothiocyanate/propidium iodide (Annexin V–FITC/PI) apoptosis detection kit (C1062S, Biyuntian, China). Cells from each group were trypsinized with 0.30% trypsin, centrifuged at 1000 rpm for 10 min, and washed with phosphate-buffered saline (PBS). The cells were then suspended in 200 μL of binding buffer containing V-FITC (10 μg/mL) and PI (5 μg/mL), incubated in the dark at room temperature for 15 min, and analyzed for apoptotic cells using the FlowJo software (version 10.7.1) [66].

### Wound healing assay

MUM-2B cells were seeded into 24-well plates containing serum-free Dulbecco’s Modified Eagle Medium/Nutrient Mixture F-12 (DMEM/F-12; Prunus, PM150310) for migration assessment. Upon reaching confluence, a uniform scratch was introduced into the cell monolayer using a sterile 200 μL pipette tip. After rinsing to remove detached cells, cultures were maintained in serum-free medium for 24 h. Images of the wound area were captured, and cell migration was quantified utilizing ImageJ software (version 1.53) by calculating the percentage of wound closure relative to the initial scratch area [67].

### Transwell assay

Following 48 h of treatment, cell invasion capability was evaluated using Transwell inserts pre-coated with 50 μL Matrigel (354234, BD Biosciences, USA), which was allowed to polymerize at 37 °C for 30 min. After rinsing with serum-free medium, cells were adjusted to a density of 2.5 × 10⁴ cells/mL, and 100 μL of cell suspension was seeded into the upper chamber. The lower chamber received 500 μL of RPMI-1640 medium enriched with 10% FBS. After 24 h of incubation, non-invading cells were removed from the upper side of the membrane with a cotton swab. The invaded cells on the lower surface were fixed using 4% paraformaldehyde (158127, Sigma, USA) for 30 min and stained with 0.1% crystal violet (C0775, Sigma, USA). Stained cells were imaged in five randomly selected fields under an inverted microscope (IXplore Pro, Olympus, Japan), and the number of invaded cells was quantified. Each experiment was performed in triplicate [68].

### Immunofluorescence staining for γ-H2AX expression

Cells were rinsed with cold PBS and fixed in 4% paraformaldehyde (P885233, Macklin, USA) for 15–30 min. Permeabilization was performed using 0.1% Triton X-100 (L885651, Macklin, USA) for 15 min. After two PBS washes, cells were incubated overnight at 5 °C in PBS containing 15% FBS. The cells or tissues were then incubated with rabbit anti-MDM2 (ab259265, Abcam, UK; 1:100) or mouse anti-γ-H2AX (ab26350, Abcam, UK; 1:100) antibodies and kept at 4 °C overnight. After washing with TBST (TBS containing 1% Tween-20) three times, the cells were incubated at room temperature for 2 h with either monkey anti-rabbit Alexa Fluor™ 594 secondary antibody (ab150075, Abcam, UK; 1:100) or goat anti-mouse FITC secondary antibody (ab6785, Abcam, UK; 1:100). Subsequently, DAPI (D1306, Thermo Fisher, USA) was used for counterstaining and fluorescence intensity was observed using a fluorescence microscope (Zeiss Observer Z1, Germany). Target areas were selected in the images for fluorescence intensity measurement. ImageJ software (version 1.53) was utilized for image processing and quantitative analysis to calculate the number of positive cells [69].

### Statistical analysis

All experimental data were derived from at least three independent replicates and expressed as the mean ± standard deviation (SD). Differences between two groups were analyzed using an unpaired two-tailed Student’s *t*-test. For comparisons involving more than two groups, one-way analysis of variance followed by Tukey’s Honestly Significant Difference post hoc test was applied when appropriate. For datasets that did not satisfy the assumptions of normality or equal variance, nonparametric tests such as the Mann–Whitney U test or Kruskal–Wallis H test were used. Statistical analyzes were performed using GraphPad Prism version 9 (GraphPad Software, Inc.) and R (version 9.0). A *p*-value < 0.05 was considered indicative of statistical significance.

## Supplementary information

Supplemental Figures

western blot

## Data Availability

The datasets generated and/or analyzed during the current study are available in the manuscript and supplementary materials.
