# Supplementary material for: High-throughput strategy for targeting MDM2 in uveal melanoma to reverse radiation therapy resistance
Source: Cell Death Discov. 2026 Apr 11;12:221. doi: 10.1038/s41420-026-02970-x (PMC13180978; doi:10.1038/s41420-026-02970-x)
Supplement: Supplementary file 1 — Supplemental Figures [file 41420_2026_2970_MOESM1_ESM.docx]

**
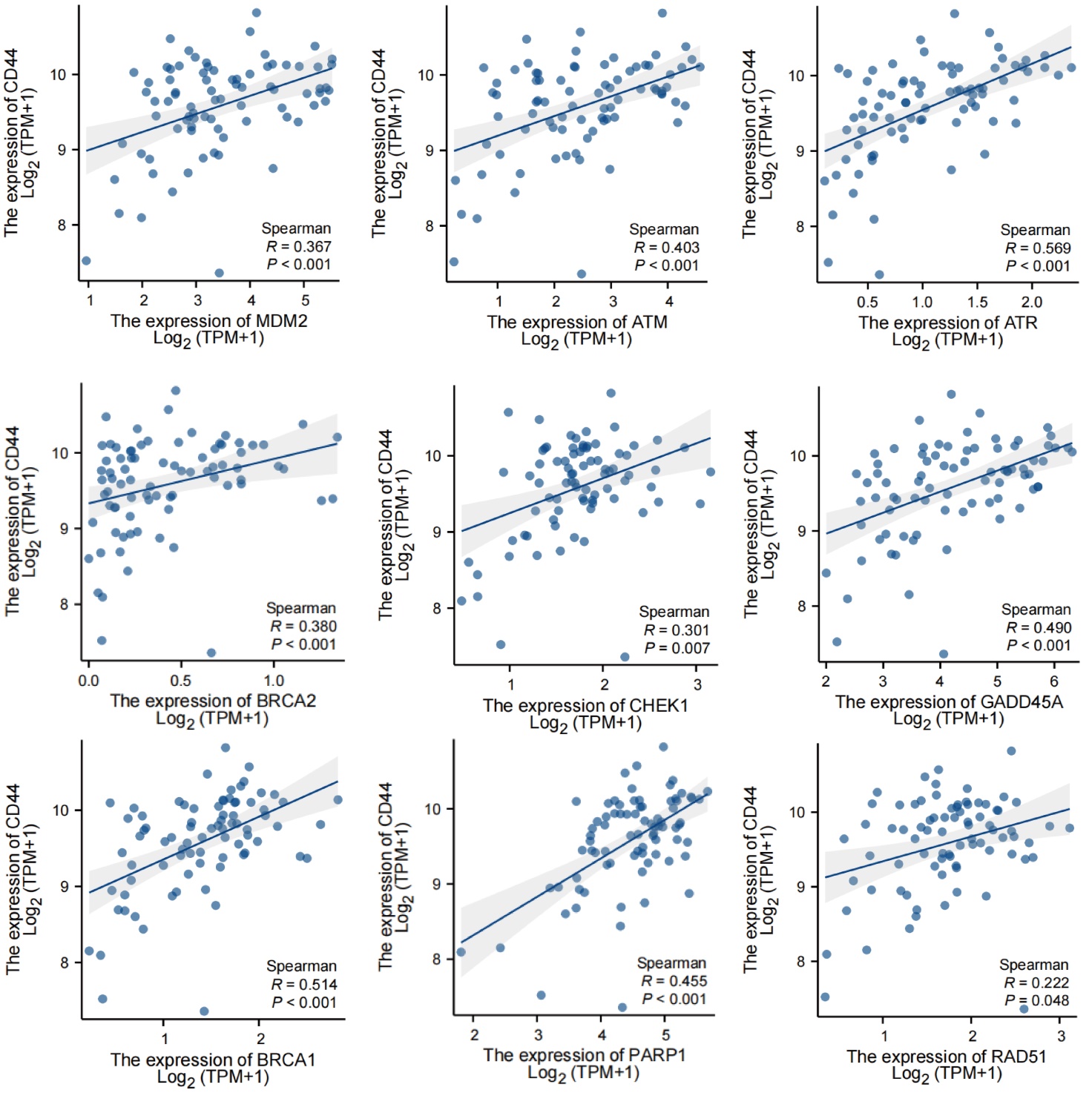
**

**Figure S1. Correlation analysis between CD44 and key DNA damage response genes in the TCGA-UVM cohort.**

Note: Transcriptomic data from the TCGA-UVM cohort were used to analyze the expression correlation between CD44 and multiple DNA damage response (DDR) core genes, including ATM, ATR, CHEK1, RAD51, BRCA1, BRCA2, PARP1, GADD45A, and MDM2. Spearman correlation analysis was conducted.

**
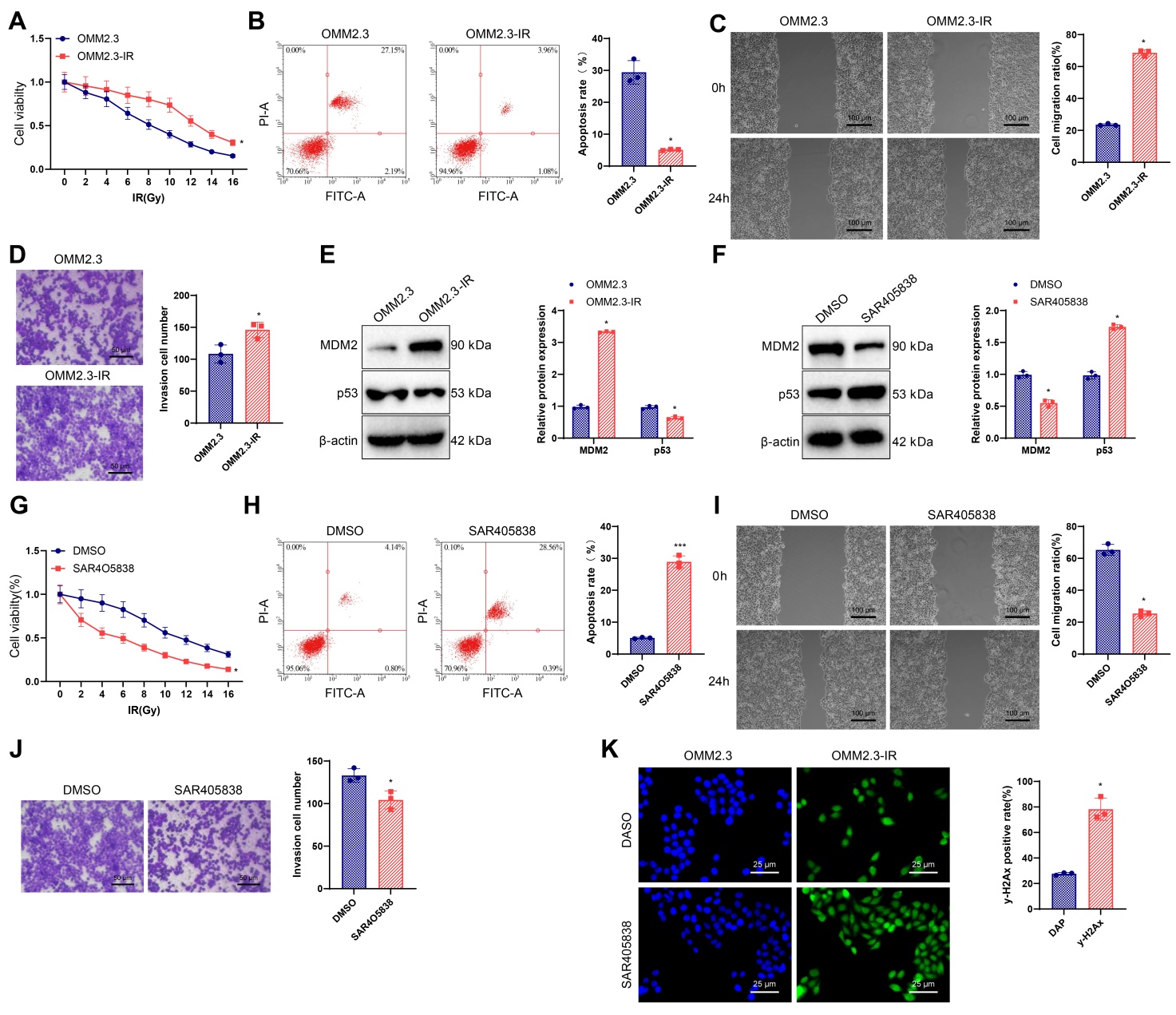
**

**Figure S2. Effects of MDM2 inhibition on reversing radiotherapy resistance in OMM2.3 cells.**

Note: (A) CCK-8 assay for cell viability of OMM2.3 and OMM2.3-IR cells under different radiation doses; (B) Flow cytometry analysis of apoptosis in OMM2.3 and OMM2.3-IR cells; (C) Wound healing assay for migration ability; (D) Transwell invasion assay; (E) Western blot detection of MDM2 and p53 expression in OMM2.3 and OMM2.3-IR cells; (F) Western blot analysis of MDM2 and p53 expression in different treatment groups of OMM2.3-IR cells; (G) CCK-8 assay for radiation-induced viability in OMM2.3-IR cells; (H) Flow cytometry analysis of apoptosis in OMM2.3-IR cells across treatment groups; (I) Wound healing assay for cell migration; (J) Transwell assay for invasion; (K) Immunofluorescence staining for γ-H2AX expression in OMM2.3-IR cells. * indicates *p* < 0.05 compared to OMM2.3 or DMSO group; all cellular experiments were independently repeated three times.
