# Supplementary material for: High-throughput strategy for targeting MDM2 in uveal melanoma to reverse radiation therapy resistance
Source: Cell Death Discov. 2026 Apr 11;12:221. doi: 10.1038/s41420-026-02970-x (PMC13180978; doi:10.1038/s41420-026-02970-x)
Supplement: Supplementary file 2 — western blot [file 41420_2026_2970_MOESM2_ESM.docx]

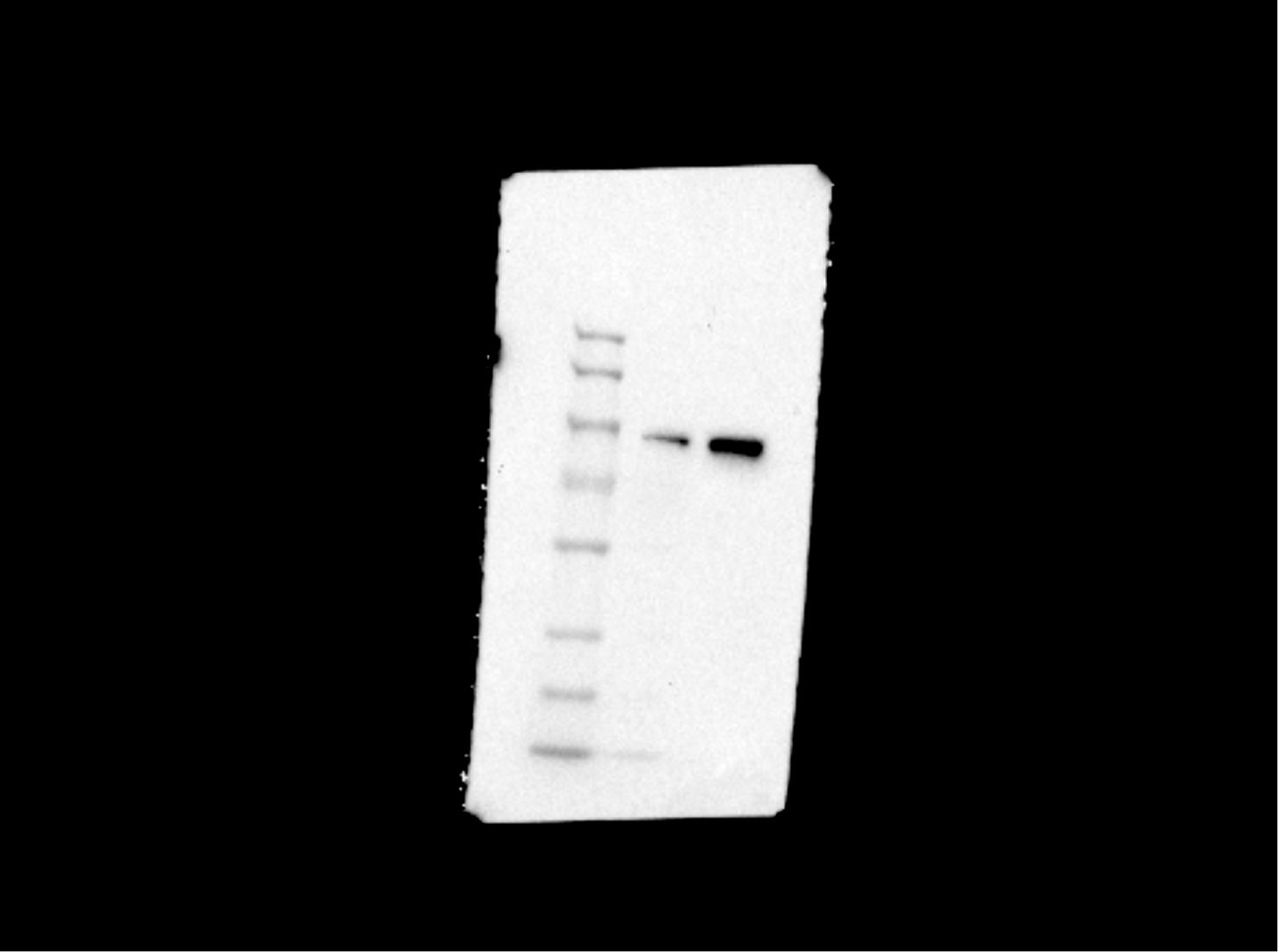


Supplementary Figure 2E-1


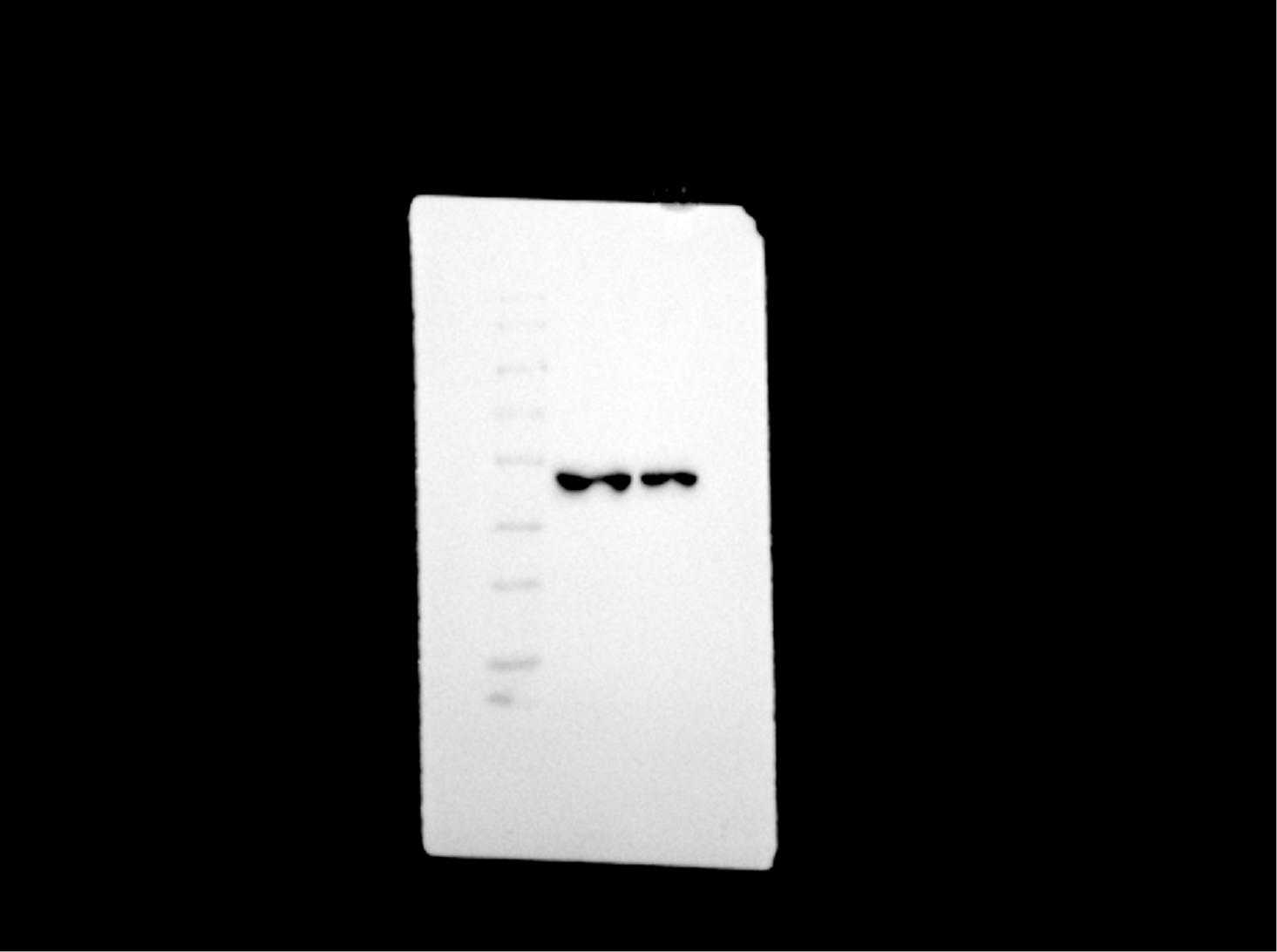


Supplementary Figure 2E-2


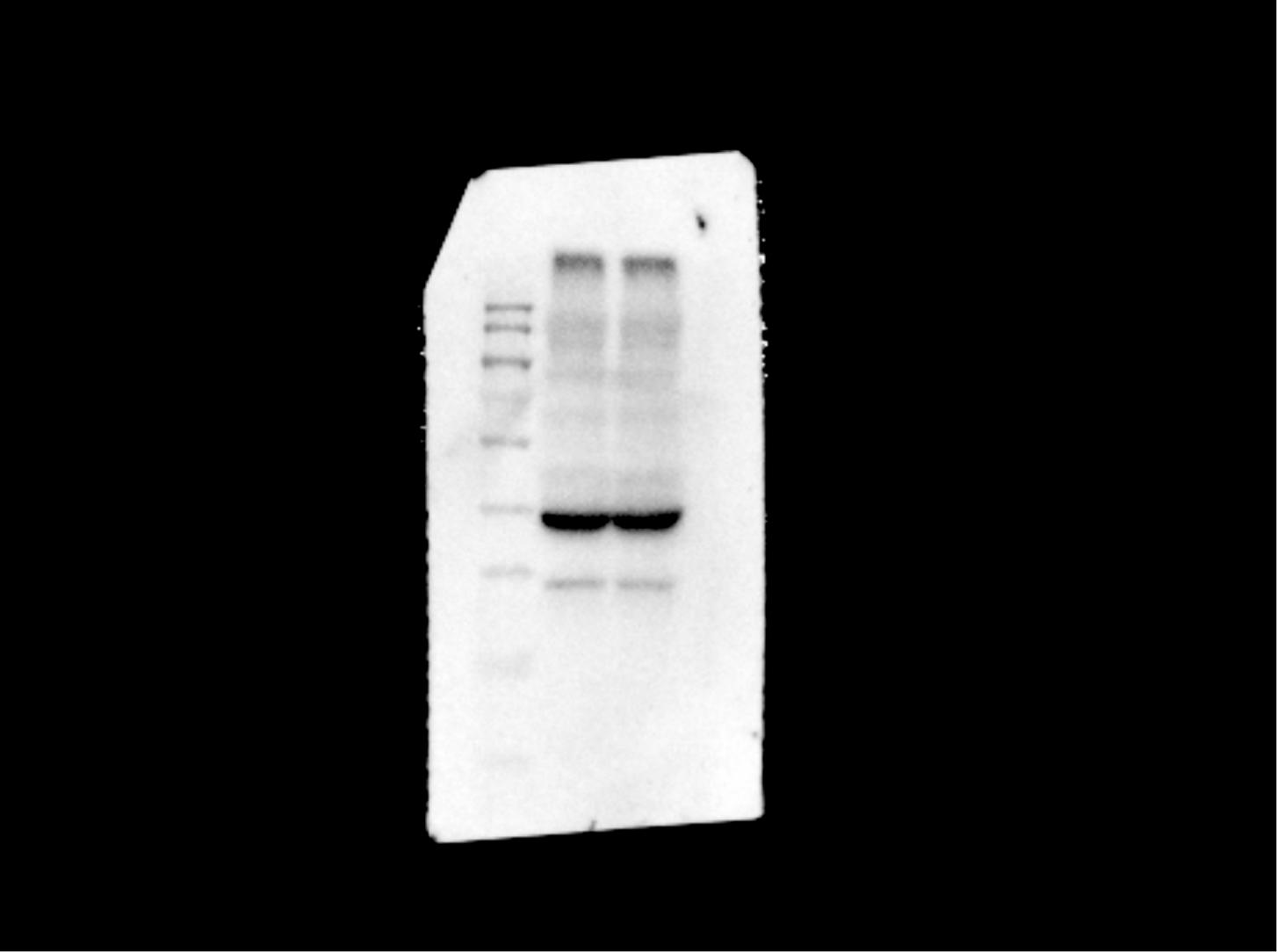


Supplementary Figure 2E-3


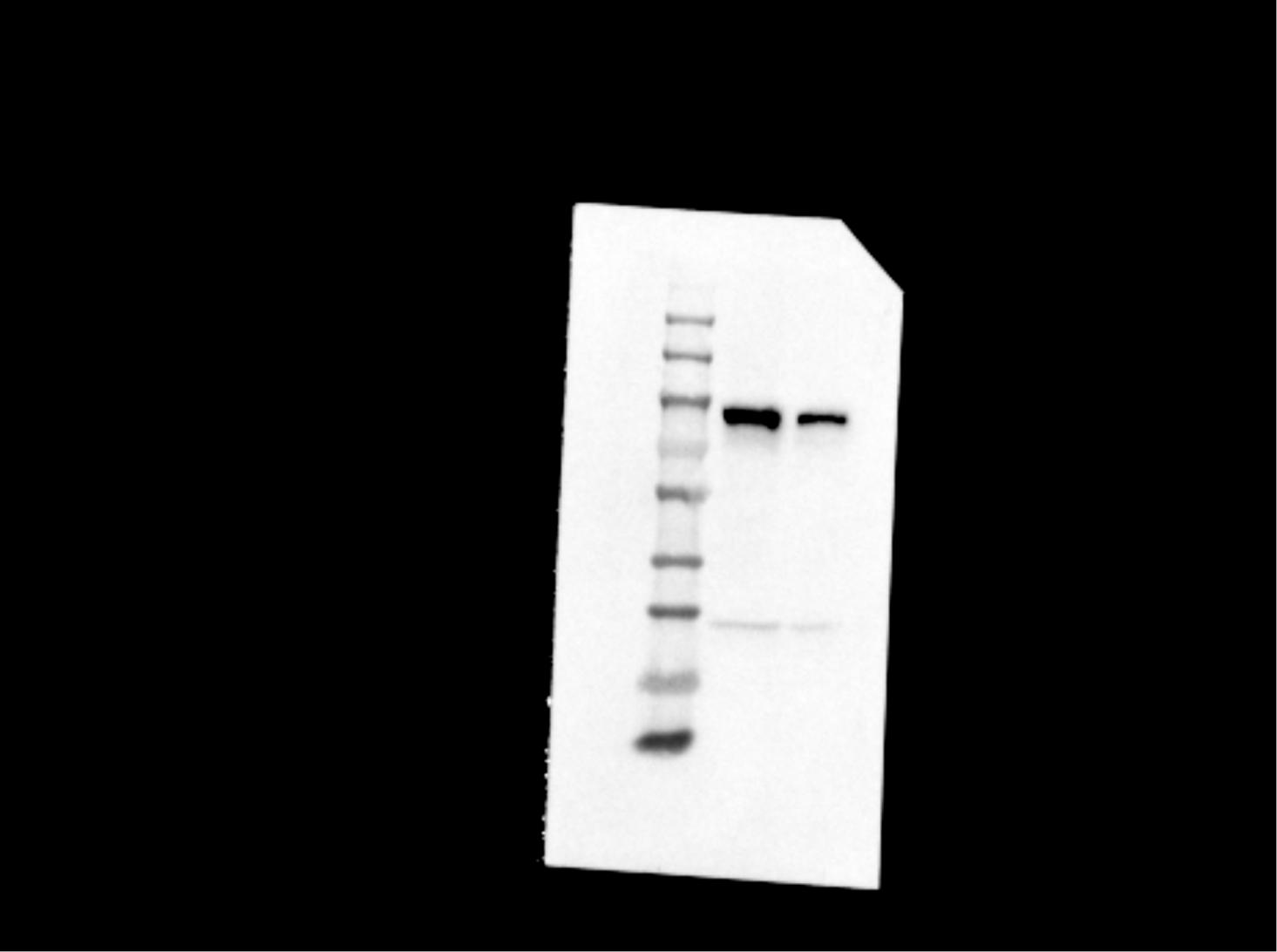


Supplementary Figure 2F-1


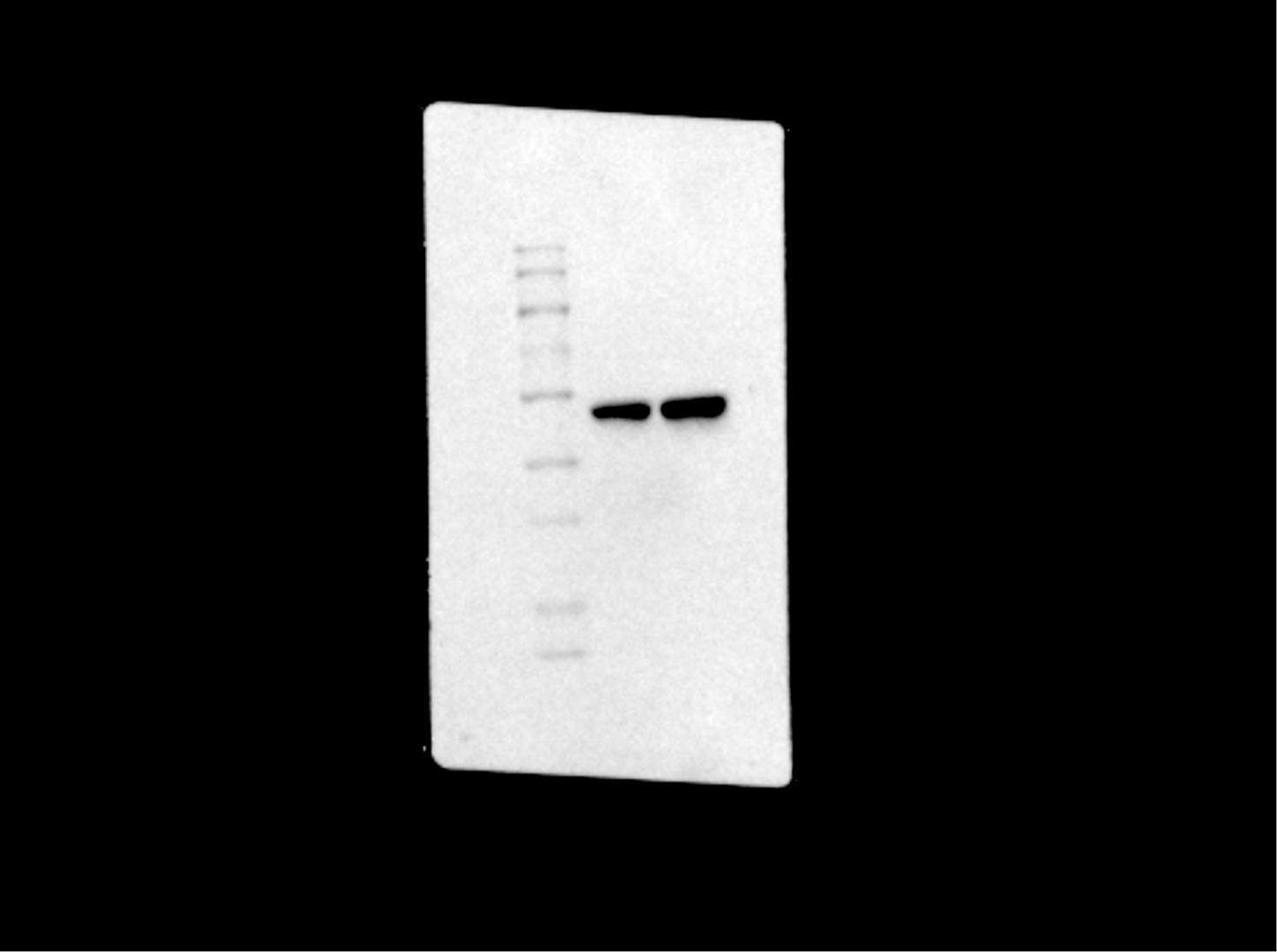


Supplementary Figure 2F-2


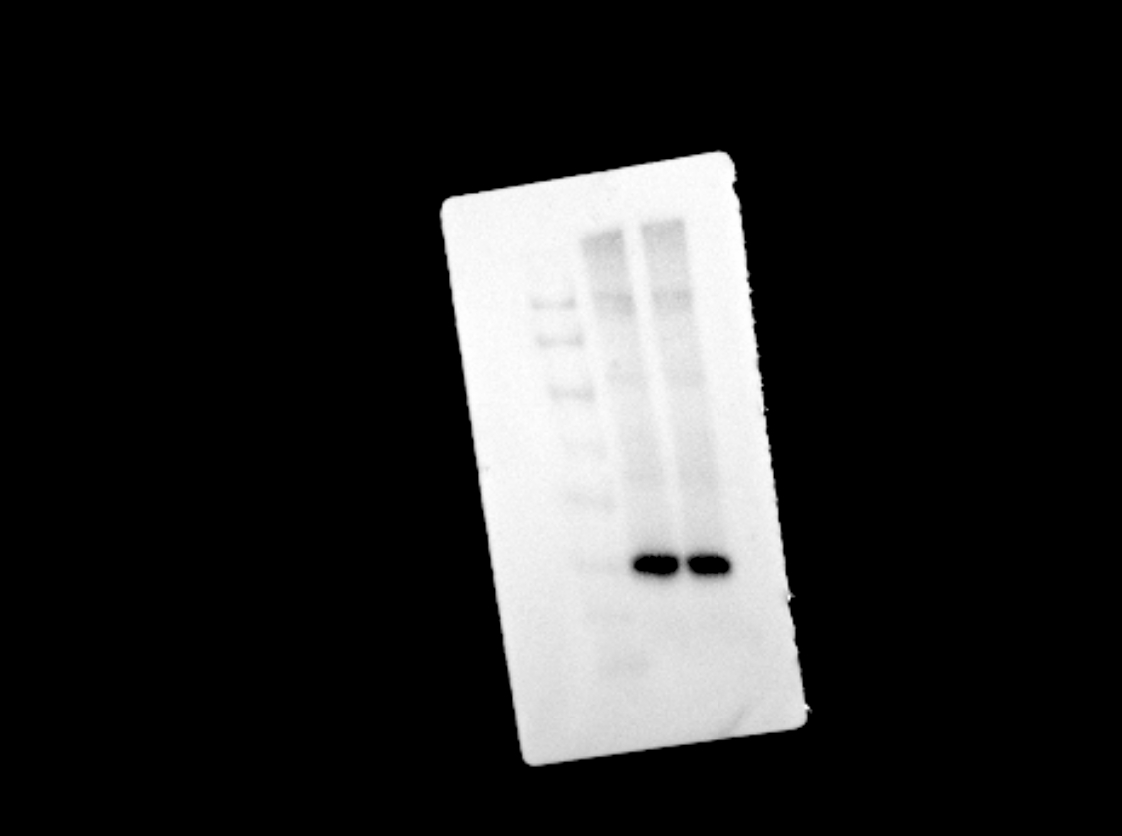


Supplementary Figure 2F-3


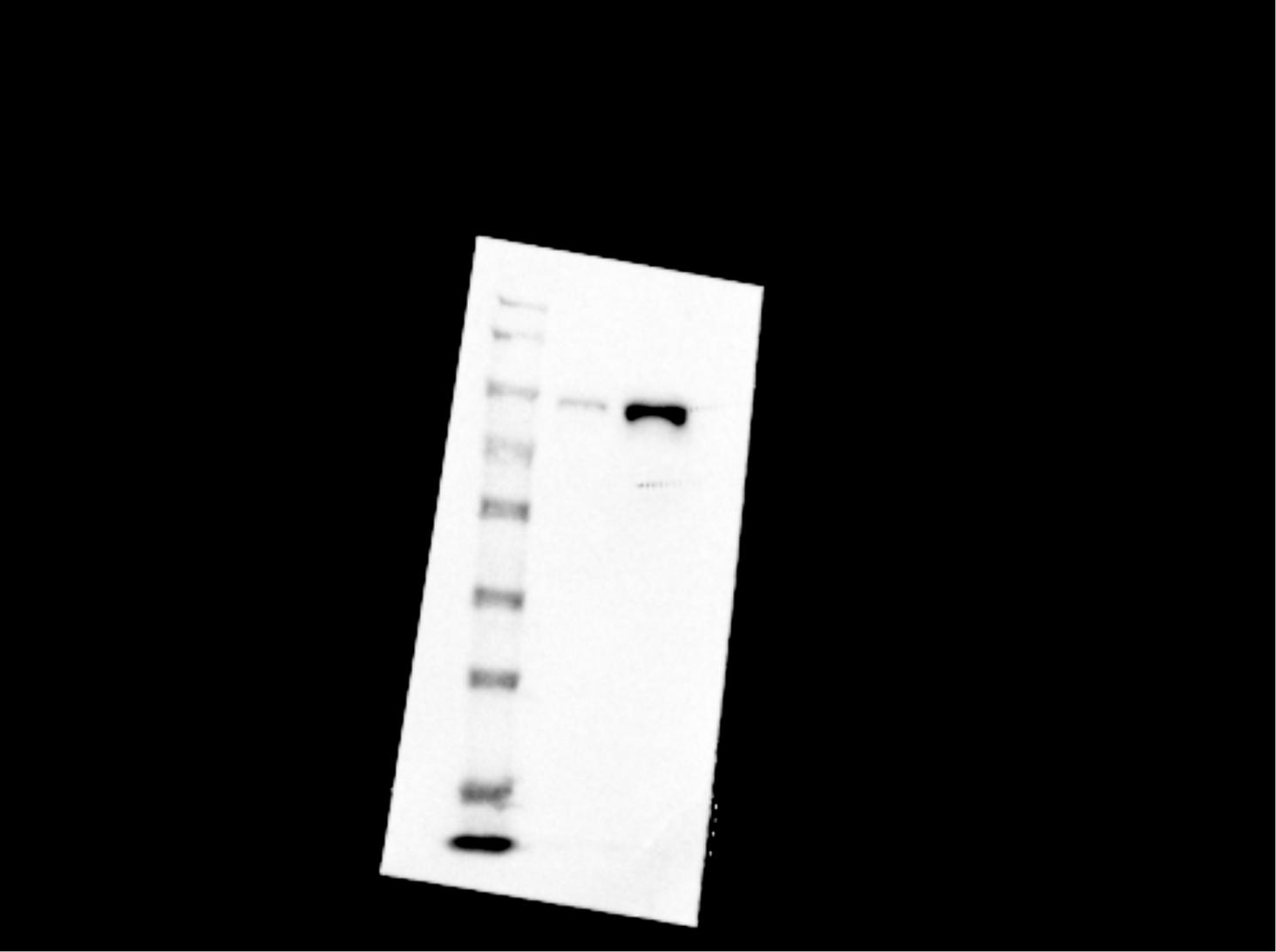


Figure 5E-1


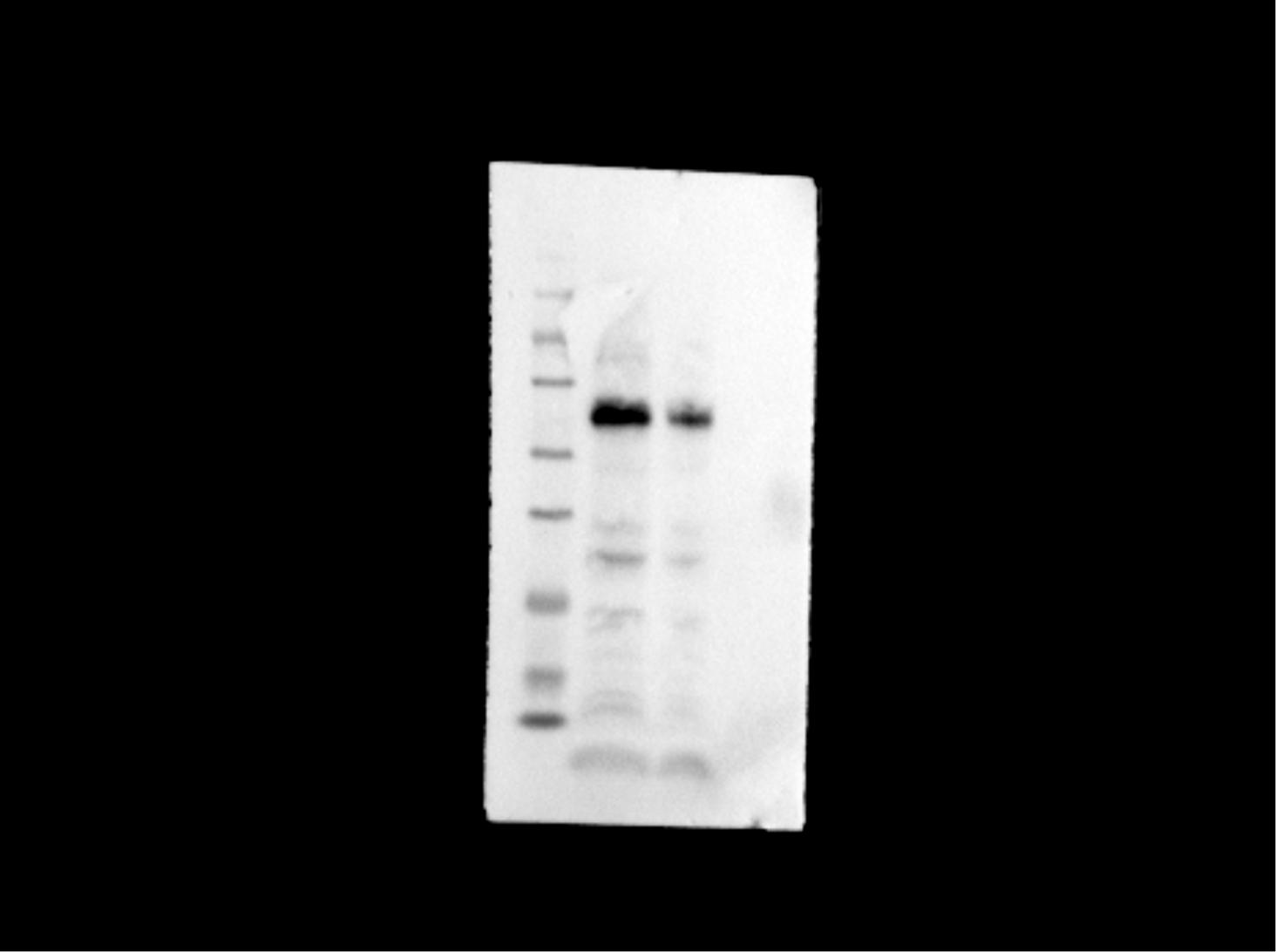


Figure 5E-2


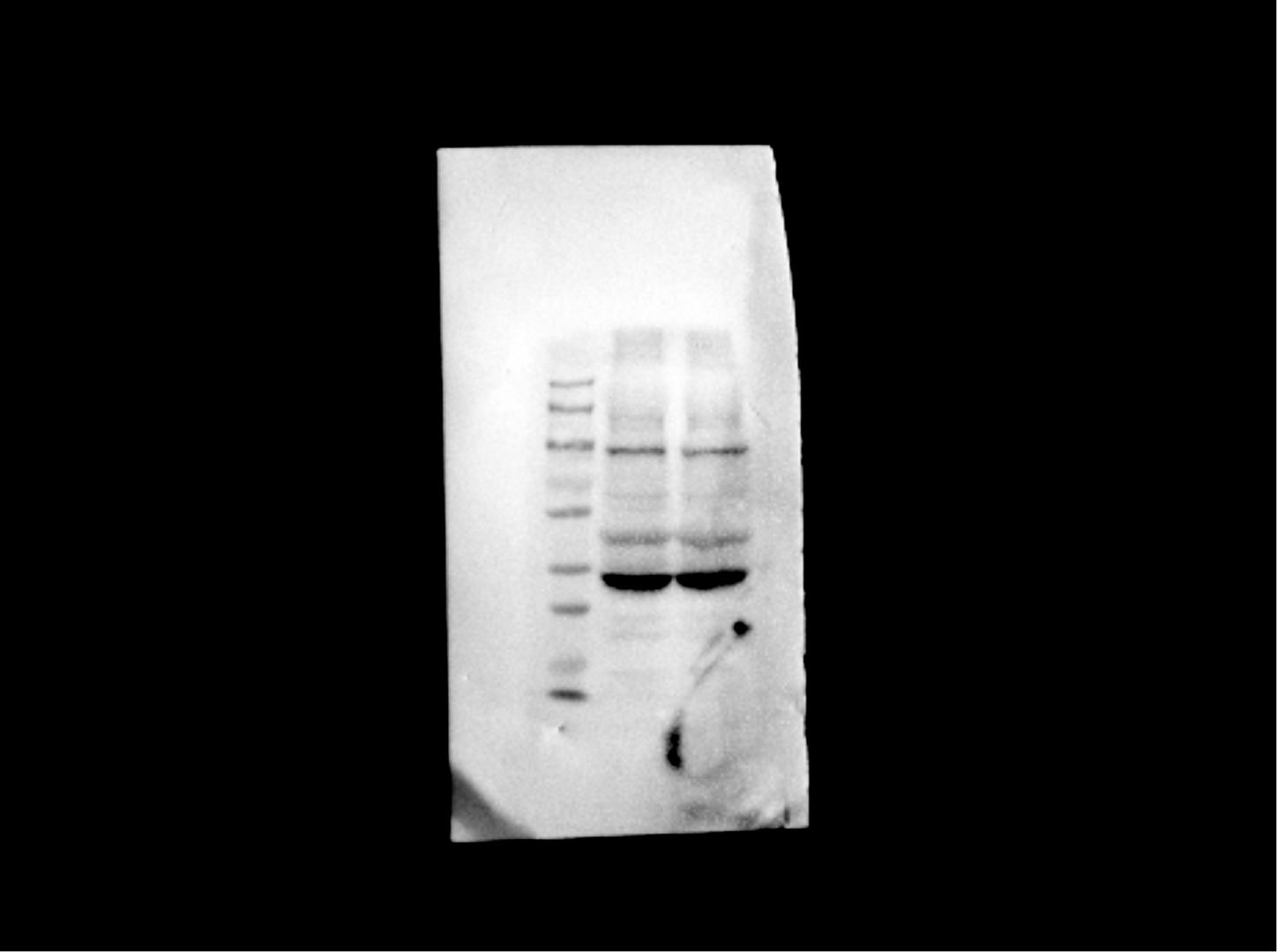


Figure 5E-3


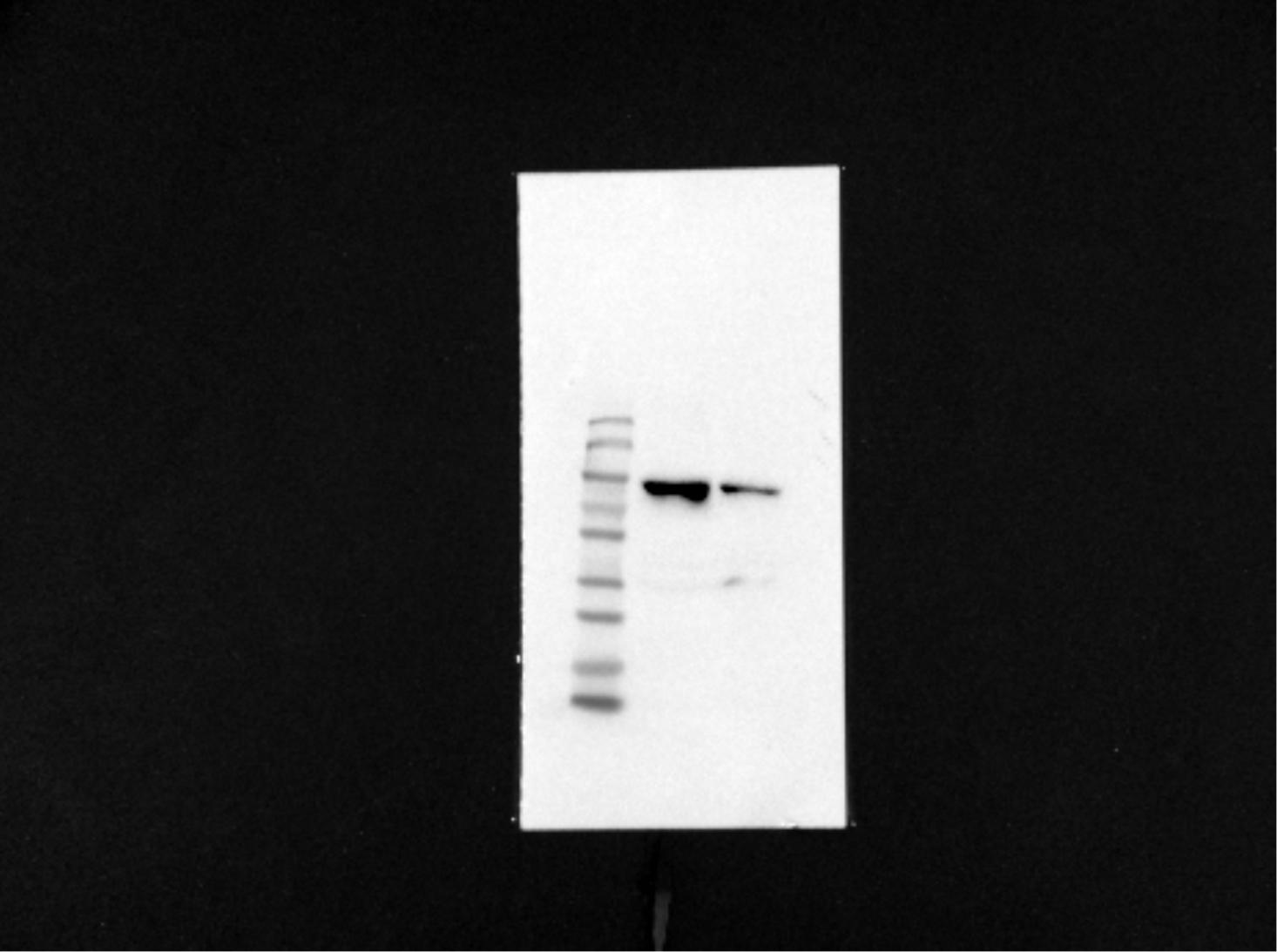


Figure 5F-1


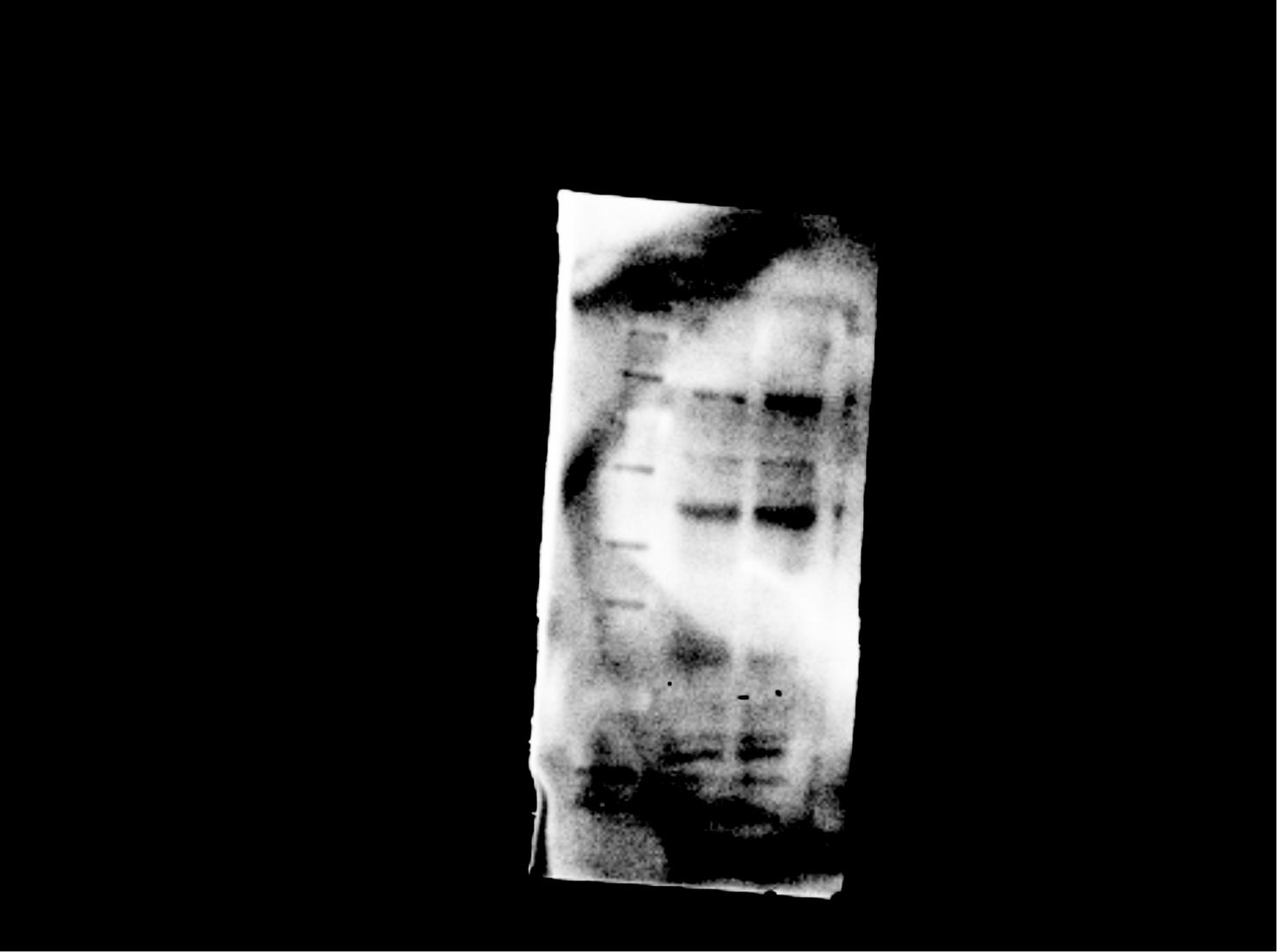


Figure 5F-2


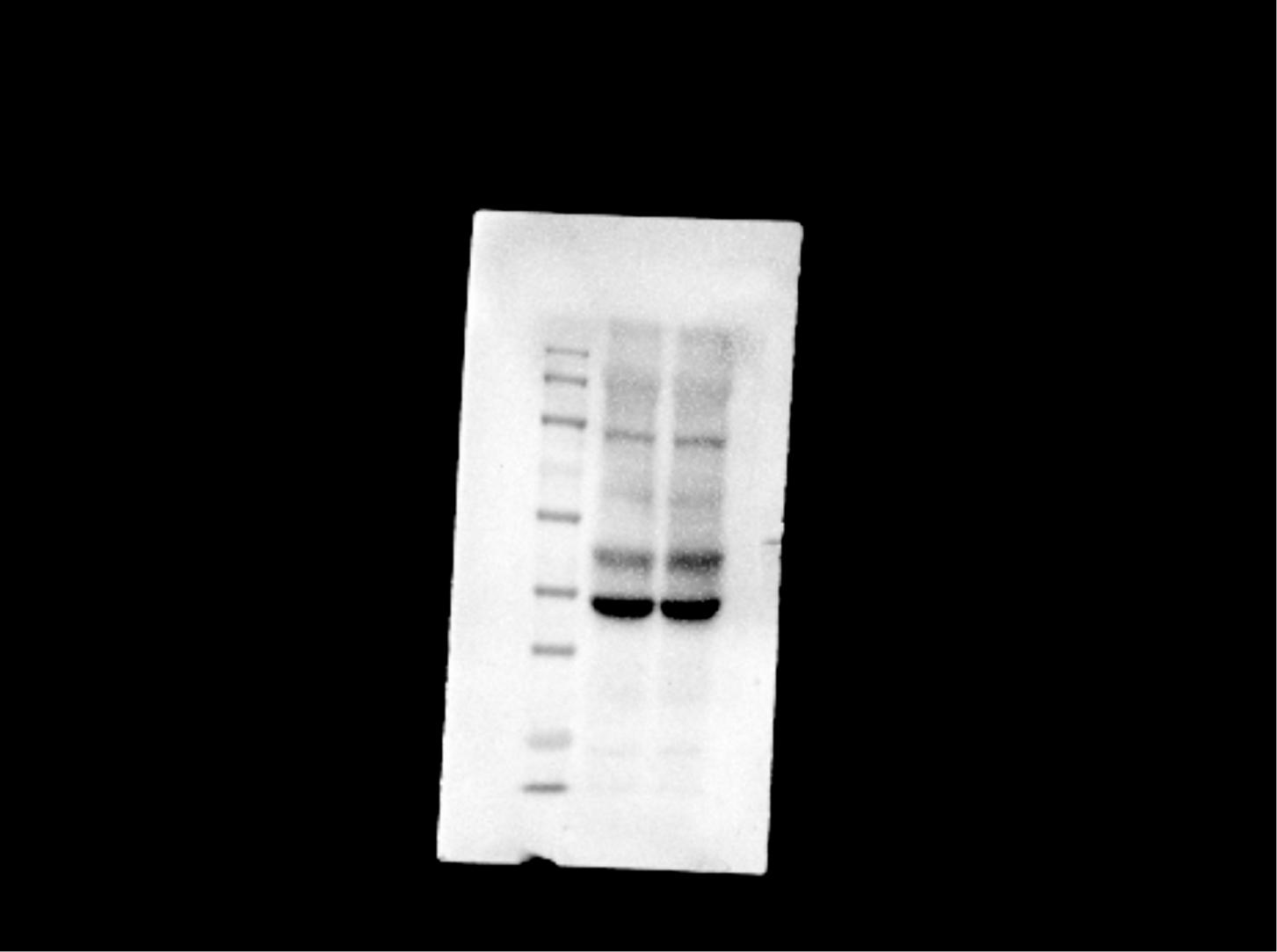


Figure 5F-3
